# Rollout of Closed-Loop Technology to Pregnant Women with Type 1 Diabetes: Healthcare Professionals' Views About Potential Challenges and Solutions

**DOI:** 10.1089/dia.2022.0479

**Published:** 2023-03-31

**Authors:** David Rankin, Ruth I. Hart, Barbara Kimbell, Katharine Barnard-Kelly, Anna Brackenridge, Caroline Byrne, Corinne Collett, Anna R. Dover, Sara Hartnell, Katharine F. Hunt, Tara T.M. Lee, Robert S. Lindsay, David R. McCance, Alastair McKelvey, Gerry Rayman, Rebecca M. Reynolds, Eleanor M. Scott, Sara L. White, Roman Hovorka, Helen R. Murphy, Julia Lawton

**Affiliations:** ^1^ Usher Institute, Medical School, University of Edinburgh, Edinburgh, United Kingdom.; ^2^ R&D, Moor Green Hospital, Southampton, United Kingdom.; ^3^ Department of Diabetes and Endocrinology, Guy's and St. Thomas' NHS Foundation Trust, London, United Kingdom.; ^4^ Cambridge University Hospitals NHS Foundation Trust, Cambridge, United Kingdom.; ^5^ Norwich Clinical Trials Unit, Norwich Medical School, University of East Anglia, Norwich, United Kingdom.; ^6^ Edinburgh Centre for Endocrinology and Diabetes, Royal Infirmary of Edinburgh, Edinburgh, United Kingdom.; ^7^ Diabetes Research Offices, Weston Education Centre, King's College Hospital NHS Foundation Trust, London, United Kingdom.; ^8^ Norwich Medical School, University of East Anglia, Norwich, United Kingdom.; ^9^ Norfolk and Norwich University Hospital NHS Foundation Trust, Norwich, United Kingdom.; ^10^ Institute of Cardiovascular and Medical Sciences, University of Glasgow, Glasgow, United Kingdom.; ^11^ Regional Centre for Endocrinology and Diabetes, Royal Victoria Hospital Belfast, Belfast, Northern Ireland.; ^12^ The Diabetes Centre, Ipswich Hospital, East Suffolk and North Essex Foundation Trust, Ipswich, United Kingdom.; ^13^ Centre for Cardiovascular Science, University of Edinburgh, Edinburgh, United Kingdom.; ^14^ Leeds Institute of Cardiovascular and Metabolic Medicine, University of Leeds, Leeds, United Kingdom.; ^15^ Department of Women and Children's Health, King's College London, London, United Kingdom.; ^16^ Wellcome Trust-MRC Institute of Metabolic Science, University of Cambridge, Cambridge, United Kingdom.; ^17^ Department of Paediatrics, University of Cambridge, Cambridge, United Kingdom.

**Keywords:** Type 1 diabetes, Closed-loop system, Continuous glucose monitoring, Pregnancy, Healthcare professional, Qualitative research

## Abstract

**Aims:**

To explore healthcare professionals' views about the training and support needed to rollout closed-loop technology to pregnant women with type 1 diabetes.

**Methods:**

We interviewed (*n* = 19) healthcare professionals who supported pregnant women using CamAPS FX closed-loop during the Automated insulin Delivery Amongst Pregnant women with Type 1 diabetes (AiDAPT) trial. Data were analyzed descriptively. An online workshop involving (*n* = 15) trial team members was used to inform recommendations. Ethics approvals were obtained in conjunction with those for the wider trial.

**Results:**

Interviewees expressed enthusiasm for a national rollout of closed-loop, but anticipated various challenges, some specific to use during pregnancy. These included variations in insulin pump and continuous glucose monitoring expertise and difficulties embedding and retaining key skills, due to the relatively small numbers of pregnant women using closed-loop. Inexperienced staff also highlighted difficulties interpreting data downloads. To support rollout, interviewees recommended providing expert initial advice training, delivered by device manufacturers together with online training resources and specific checklists for different systems. They also highlighted a need for 24 h technical support, especially when supporting technology naive women after first transitioning onto closed-loop in early pregnancy. They further recommended providing case-based meetings and mentorship for inexperienced colleagues, including support interpreting data downloads. Interviewees were optimistic that if healthcare professionals received training and support, their long-term workloads could be reduced because closed-loop lessened women's need for glycemic management input, especially in later pregnancy.

**Conclusions:**

Interviewees identified challenges and opportunities to rolling-out closed-loop and provided practical suggestions to upskill inexperienced staff supporting pregnant women using closed-loop. A key priority will be to determine how best to develop mentorship services to support inexperienced staff delivering closed-loop.

Clinical Trials Registration: NCT04938557.

## Introduction

Pregnant women with type 1 diabetes (T1D) are advised to aim for target glucose levels of 3.5–7.8 mmol/L for at least 70% of the day.^1^ Most women experience difficulties attaining these targets.^2,3^ Alongside challenges calculating carbohydrates and determining insulin dosages, physiological changes result in periods of insulin sensitivity in early pregnancy followed by increasing insulin resistance as pregnancy progresses.^4^ To help optimize glycemic management in pregnancy, guidelines recommend that women receive input and support from joint diabetes/antenatal teams every 1–2 weeks.^5^

Closed-loop systems have begun to transform T1D management.^6^ These systems combine a real-time continuous glucose monitor (CGM) with an insulin pump and an algorithm that automatically adjusts insulin delivery based on sensor glucose levels. To date, four hybrid closed-loop (HCL) systems are used commercially with regulatory approval in the United States and Europe,^7^ with further systems under development.^8^ The CamAPS FX platform, which includes customizable glucose targets suitable for optimal pregnancy glucose targets, is the only system currently licensed for use in T1D pregnancy.^9^

Healthcare professionals play pivotal roles in mediating people's expectations, experiences, and use of diabetes technologies.^10–15^ Hence, to support pregnant women using HCL, it is important that their perspectives are considered. To date, interview studies have focused on healthcare professionals' experiences of supporting adolescents using HCL,^14,16^ or have consulted those with limited knowledge and first-hand experience of using HCL in T1D pregnancy.^12,17^

To address this gap in the literature and provide bespoke recommendations for supporting HCL use in pregnancy, we report findings from interviews with healthcare professionals who participated in a UK-based, multicenter, randomized controlled trial—Automated insulin Delivery Amongst Pregnant women with Type 1 diabetes (AiDAPT). The trial, which began in September 2019 and closed out in November 2022, explored the biomedical and psychosocial effects of using HCL (CamAPS FX) compared with CGM in pregnant women (aged 18–45 years) receiving routine clinical care.^18^ For details of the HCL used, see Box 1. Women randomized to HCL used the CamAPS FX system from early pregnancy (∼12 weeks) until delivery (∼36–38 weeks).

**Box 1.** The Closed-Loop System Used During the Trial

The CamAPS FX system combines real-time CGM (CGM), the Dexcom G6 CGM sensor and transmitter (Dexcom, San Diego, CA) with an insulin pump, the DANA RS (Sooil, Seoul, South Korea), via the Cambridge model predictive control algorithm (CamDiab, Cambridge, UK), hosted on an unlocked Android smartphone (Galaxy S7–S10, Samsung, South Korea), running Android 8 OS or above.
The smartphone/app communicates wirelessly with the CGM sensor and insulin pump, subject to being kept within 5–10 meters range of both devices. It uses CGM sensor data to direct (basal/background) insulin delivery via the pump, adjusting this automatically every 8–12 min. As well as being used to administer premeal bolus doses, the CamAPS FX app is used to (1) set personal glucose targets, typically 5.5 mmol/L (99 mg/dL) in early pregnancy and 5.0 mmol/L (90 mg/dL) after 14–16 weeks, consistent with achieving and maintaining pregnancy glucose targets; (2) adjust the rate of insulin delivery using ‘Boost’ and ‘Ease-off settings’; (3) personalize alarms to alert about high/low glucose levels; and (4) view ‘real-time’ glucose levels, rate of insulin delivery and summary statistics.
*Remote monitoring capabilities*
The app automatically uploads data to the cloud (Glooko/Diasend; Göteborg, Sweden), enabling data sharing with other individuals, including healthcare professionals and partners/family members. Using the CamAPS FX clinical portal, healthcare professionals at local sites and members of the trial team could access the following data:
• ‘Real-time’ and retrospective graphs displaying CGM and capillary blood glucose levels, rate of insulin delivery, insulin boluses, and carbohydrate intake, high/low glucose range, Boost and Ease-off status, and system status (operational or interrupted/switched off).
• Summary statistics (daily, weekly, monthly, or three-monthly periods), including mean CGM glucose, GMI or estimated HbA1c, time in/below/above target glucose range, number and average duration of hypos, total daily dose/bolus/basal insulin, and percentage of time in operation.
Healthcare professionals could also receive summary CamAPS FX reports by email, daily, weekly, or monthly, for participants using the HCL at their site. These included key glycemic metrics (mean glucose; time in/above/below target glucose range), insulin doses, and system metrics (HCL use, CGM use, and number of alarms issued during the day and at night).

CGM, continuous glucose monitor; GMI, Glucose Management Indicator; HbA1c, glycated hemoglobin; HCL, hybrid closed-loop.

Healthcare professionals at local sites, working alongside a research educator, trained women to use the system, provided study-related support, and were responsible for participants' routine clinical care. Due to the Covid-19 pandemic, research visits and device training were available virtually via video call or telephone, and most participants' diabetes appointments were also offered virtually, mirroring practices in routine clinical care. For further details regarding the AiDAPT trial and training provided, see Box 2.

**Box 2.** Details About the Automated Insulin Delivery Amongst Pregnant Women with Type 1 Diabetes Trial and Training Provided

*AiDAPT trial*
The AiDAPT trial was designed to evaluate the clinical efficacy of HCL insulin delivery throughout pregnancy (∼24 weeks duration, from 14 to 38 weeks) in real-life NHS maternity care settings (see: https://www.isrctn.com/ISRCTN56898625).
To be eligible for the trial, women had to have been diagnosed with T1D for at least 12 months, have a viable pregnancy confirmed by ultrasound (up to 13 weeks and 6 days duration), be using intensive insulin therapy (MDI or pump, including sensor-augmented pumps or other HCL system), be able to wear and use study devices, and have an HbA1c level ≥48 mmol/mol at booking (first antenatal contact) and ≤86 mmol/mol (at randomization). Women with an HbA1c <48 mmol/mol were considered to be optimally managed and achieving glucose targets as per current recommendations.^20^ These women were not included in the trial as it was felt that they would be unlikely to derive significant glycemic benefit from using HCL technology.
Following randomization, participants in the intervention arm received training to use the CGM, insulin pump, and CamAPS FX app. Due to the Covid-19 pandemic, participants in both study arms were given the option to attend training (and research visits) virtually via video-call or telephone, with virtual support provided by a research educator. Initiation on the HCL included starting/stopping the system, meal bolus procedure, use of alarms, and device trouble-shooting. Staff at local sites assessed women's competency to use the HCL. Training recommendations were reinforced using pregnancy ‘top tips' leaflets, mealtime and CGM advice (https://abcd.care/dtn-uk-top-tips); and webinars (https://camdiab.cdept.org.uk/). Participants had access to a 24 h telephone helpline to contact their local study team with any study-related matters. They could also contact the research educator if requiring technical support.
The primary outcome is the percentage of time spent with CGM glucose levels between 3.5 and 7.8 mmol/L between 16 weeks' gestation and delivery. Secondary outcomes include maternal glycemic, obstetric, and psychosocial outcomes, neonatal health outcomes, safety outcomes, healthcare professional experiences, and health economic outcomes. Further detail about the trial is available in the study protocol.^18^
*Training for healthcare professionals*
The trial was conducted in nine UK sites involving healthcare professionals who had varying levels of experience supporting diabetes technologies, ranging from those who had experience of both CGM and insulin pumps to those with none. Changes to NICE guidelines^5^ meant that all sites began using Freestyle Libre during 2020, followed by CGM in 2021. Healthcare professionals not familiar with the Dana insulin pump and Dexcom G6 CGM were encouraged to attend training with representatives from the device manufacturers before their site start.
The research educator provided training on the HCL's individual components for the first participants at each study site and as required, thereafter. The initial HCL training session at each site was used as a training opportunity for the local clinical team, delivered by the research educator either face-to-face or virtually (depending on site location and Covid-19 restrictions). If there were long gaps between HCL participants, or if staffing was difficult due to Covid-19 redeployments, the research educator delivered the training either in full or by involving the local clinical team as a refresher. Competency checklists were used for each system component to ensure all aspects of the training had been covered.
Additional resources used to support training included electronic information sheets (Top Tips for using CGM or closed-loop during pregnancy); peer support from experienced colleagues and case-based discussions to share healthcare team experiences; and publicly available webinars and CamDiab online training (https://www.camdiabtraining.com/) on utilizing the CamAPS FX HCL during pregnancy. The CamAPS FX clinic portal allowed HCL users' glucose data to be aggregated for review (daily, weekly, or monthly) by local clinical teams and/or by members of the research team. There is also an in-app link to the user manual, which includes trouble-shooting tips and Frequently Asked Questions. In addition to delivering initial HCL training, the research educator provided ongoing technical support for sites, as required.

AiDAPT, Automated insulin Delivery Amongst Pregnant women with Type 1 diabetes; MDI, multiple daily injections; T1D, type 1 diabetes.

Elsewhere, we have described how healthcare professionals favored an inclusive approach to HCL use and rollout due to their perception and understanding that virtually all pregnant women can experience clinical and/or quality-of-life benefits from using a HCL.^19^ Here, we explore healthcare professionals' views about challenges and opportunities that a national rollout could present, including their training and resource needs, to support HCL use in pregnant women in routine clinical care.

## Methods

We used an inductive, semistructured interview design, informed by topic guides to ensure that the discussion remained relevant to addressing the study aims, while allowing flexibility for participants to raise issues they perceived as salient. The study involved concurrent data collection and analysis, which allowed issues identified in early interviews to inform questions asked in later ones. Research ethics (Cambridge Central Research Ethics Committee: 18/EE/0084) and governance approvals were obtained in conjunction with approvals for the wider trial.

### Recruitment

Healthcare professionals were recruited from eight participating trial centers across the UK, after they had ≥6 months' experience of supporting women using HCL. Recruitment continued until there was good representation of different grades of staff from across the eight sites and no new findings were identified in new data collected.

### Data collection and analysis

Telephone interviews were conducted by D.R., an experienced (nonclinical) qualitative researcher between June 2021 and April 2022. Topic guides were developed in light of earlier investigations of HCL use,^14,16^ and inputs from clinical coinvestigators, were revised in response to emerging findings and used flexibly according to healthcare professionals' roles and involvement in the trial. Key topics relevant to the reporting in this article are described in Box 3. Interviews that lasted 1–2 h were digitally recorded and transcribed in full.

**Box 3.** Key Areas Explored in the Interviews

• Participants' clinical background, training, and experience; previous involvement (if any) in trials of HCL technology or supporting HCL users receiving routine care.
• Experiences (if any) of training study participants to use the HCL.
• Experiences of providing support to participants using a HCL during pregnancy; perceived differences in the type and amount of support required compared with people using conventional insulin regimens (e.g., CGM with pump and/or MDI); perceived sustainability of providing this level of support upon rollout.
• Perceived benefits and drawbacks of using HCL technology compared to other regimens used to manage T1D in pregnancy.
• Experiences of training and support received to deliver the trial; views about the kinds of training, support, and resources healthcare professionals will need to support HCL users in routine clinical care.
• Views about who should provide and how services should be organized or structured to deliver HCL therapy in routine clinical care.
• Perceived impact of rolling out HCL technology on healthcare professionals' clinical practice, workloads, and wider healthcare resources.

We undertook qualitative descriptive analysis, which focuses on identification and description of minimally theorized explanatory themes and is suited to understanding and illuminating issues relevant to policy and practice.^21,22^ To promote rigor, four experienced (nonclinical) qualitative researchers (D.R., J.L., R.I.H., and B.K.) undertook independent analyses, which involved repeatedly reading and cross-comparing individuals' interview transcripts to identify cross-cutting themes. Each researcher wrote a separate report before meeting to discuss their interpretations and agree on a coding frame that captured areas of relevance to addressing the study aims. Coded datasets were then subjected to further analyses to allow more granular insights to be developed. The qualitative analysis software package NVivo20 (QSR International, Doncaster, Australia) facilitated data coding and retrieval.

### Analytical workshop

Preliminary findings were discussed at an online workshop, held in September 2022, involving principal investigators, healthcare professionals who participated in the trial, other trial staff, and members of the qualitative research team. A ‘What? So What? Now What?’ approach was used to develop realistic and practical recommendations relevant to a range of diabetes professionals and clinical settings.^23^ Individuals who were unable to attend the meeting contributed to the generation of recommendations via email correspondence.

## Results

The sample comprised 19 healthcare professionals: 11 doctors, six diabetes specialist nurses (four nurses, two nurse consultants), one dietitian, and one diabetes midwife. See Table 1 for further details. Below, we report interviewees' accounts of the workforce and skill deficits likely to affect wider rollout of HCL in maternity clinics, their suggestions for how these might be addressed, and their views about how investing time and resources to upskill staff upfront could lead to reductions in future workloads. As responses did not differ according to professionals' roles, individual characteristics are not separated out in the reporting below. Key illustrative quotations are included below; for additional quotations, see Table 2.

**Table 1.** Participant Characteristics

	N	%
AiDAPT sites (*n* = 8)		
Total number of interviewees	19	
Interviewees per site—range (mode)	1–4 (3)	
Role^a^		
Diabetes consultants/doctors	11	57.9
Nurse consultants	2	10.5
Diabetes specialist nurses	4	21.1
Dietitian	1	5.3
Diabetes specialist midwife	1	5.3
Years of diabetes experience		
5–10 years	4	21.1
10–20 years	5	26.3
>20 years	10	52.6
Interviewees with previous experience supporting HCL users during trials or in routine care	12	63.2

^a^ Percentages do not sum to 100% due to rounding.

AiDAPT, Automated insulin Delivery Amongst Pregnant women with Type 1 diabetes; HCL, hybrid closed-loop.

### What challenges need to be addressed to support pregnant HCL users in routine clinical care?

#### Skills shortages

As several interviewees observed, after setting up and linking components, training and supporting women to use the HCL app/algorithm was “pretty straightforward” (HP-003) because “there aren't an enormous number of things to change… it seems a fairly intuitive interface” (HP-012). Given this, interviewees suggested that the greatest challenge to rolling out HCL technology to pregnant women would reside with upskilling centers and colleagues with limited experience interpreting CGM data and/or supporting insulin pump users (Table 2).

**Table 2.** Additional Participant Quotations

Themes	Subtheme and participant quotations
Challenges supporting pregnant HCL users in routine clinical care.	*Skills shortages* “I think if you have pump knowledge and CGM knowledge the closed-loop is very straightforward. [However,] pump is still not universal and so depending on the center, maybe some of the bigger centers have 30, 40% of people on pumps, some centers have only got 10% on pumps, so pump knowledge is very variable.” (HP-009) “We have a very stretched workforce and this is not top of the list of priorities. And I don't think they see it as necessarily for them [upskilling]… because they don't even have the capacity to put that effort in … If I'm worried about doing it [in a large center], imagine what smaller places … would be like … who've perhaps even got less support or are less interested.” (HP-011) “One of the challenges is making sure that enough members of the team are competent to manage it and for it not to become so specialized that only two clinicians out of the team of eight, actually know what they're doing … that we don't limit that to so few consultants, that if somebody's on holiday for a week, there's nobody who can help. I think that's quite a big challenge.” (HP-010) “Some areas of the country, patients aren't going to be able to travel… maybe in, I don't know, the Highlands of Scotland or … some places in Cornwall … are we saying that those people won't be able to access it …just because they live round the corner from (names experienced hospital) that shouldn't be the reason for access.” (HP-013) *Challenges specific to T1D pregnancy* “The average maternity clinic will have 20 women with type 1 a year. So, if they were going to put ten women on systems and they had access to three or four different systems, it's going to be hard.” (HP-014)
Healthcare professionals' training, resource and support needs to roll-out HCL	*Training resources* “Have really clear checklists about what needs to be done … how you link everything together, what the patient must do, and what you must sign off [with] the patient… so they look at the videos, they know what to do.” (HP-013) “If you've only got, let's say ten women with type 1 coming through your service, having to learn three different systems is- is really hard. So being able to say, yes, we can offer closed loop, but it will be this system, is a possibility.” (HP-003) *Infrastructure to support clinical teams to develop and consolidate local HCL expertise* “She [research educator] did the first patient and we were both there… the second one we did… but [research educator] was watching virtually. And then after that … [research educator] was there if we got stuck. So that was brilliant… That's a much better way of learning, certainly for me.” (HP-003) “I think this is similar to going onto general pumps… getting people, like reps from the company. So, like pump starts for Dana, Medtronic… and they train the women… basically like [research educator], who works for CamAPS as opposed to working for the NHS.” (HP-018) *Technical support for healthcare professionals* “You do kind of need that expert support kind of on-hand, to be able to call somebody and say: right, this is happening … tricky things like: the app's not working, the transmitter's failed, you know, I've tried this, this and this, what next?” (HP-002) “I suppose like any commercial product like Libre or Dexcom, you're going to have to have a national 24-h helpdesk, aren't you? … It's a bit like you know, if you produce an insulin pump … or provide Dexcom, you have to have a helpline, an easy, accessible, functional, user-friendly helpline. You can't just bung the technology out there and expect individual sites to be able to answer all the questions. You're providing a commercial product. … So, the manufacturer has to provide that help.” (HP-007) *Glycemic management support for healthcare professionals* “[What's] been really helpful on occasion is, where I've been able to talk to [central trial staff] about, carb ratios, and when to strengthen them and when to weaken them, and use ease-off and boost… sometimes it can get a bit hard to know which bit of the algorithm is working when.” (HP-004) “Maybe having wider support meetings if you like…. a Type 1 pregnancy technology MDT [multidisciplinary team meeting], where we discuss just a couple of cases every couple of weeks, with the wider team in more detail, so that we upskill if you like, other people in the team, who are not doing technology so much… and if you could get that between centers, being able to share that experience is really important.” (HP-003)
Short-term investment for long-term gains	“It does give you just a general brief overview… you can cast your eyes over and be like: ‘oh, they're not doing too badly, or the time in range isn't too bad. That's someone I don't need to focus on so much, whereas it might highlight someone else that you do need to focus on.” (HP-019)

T1D, type 1 diabetes.

Several noted that it would be particularly challenging to upskill healthcare professionals in smaller sites with a fluid workforce, because of staff shortages and difficulties in appointing new members who are able to support technology use. It was also noted that, in some smaller and less experienced sites in particular, existing staff lacked time or motivation to take on new training, an issue heightened by the Covid-19 pandemic (Table 2). Many also emphasized that even in experienced centers, a wider group of staff would need to be upskilled, to avoid reliance on a “subset” (HP-009) with relevant technology expertise (Table 2).

Moreover, interviewees indicated the importance of upskilling a wider corpus of hospital staff (e.g., in Accident and Emergency, Maternity Assessment and Delivery units) who provide support to women presenting acutely (e.g., with diabetic ketoacidosis): “so when patients are admitted… wider healthcare professionals aren't doing things they shouldn't be doing” (HP-015).

Several expressed additional concerns that, without widespread upskilling, existing regional disparities in healthcare professionals' skills and expertise might result in women in certain locations, especially those living far away from centers with HCL technology experience, having inequitable access (Table 2).

#### Challenges specific to T1D pregnancy

As interviewees further observed, while similar challenges would apply to upskilling an existing workforce to support HCL more generally, these were amplified in maternity clinics, where even large centers supported relatively few T1D pregnant women (e.g., ∼20 per year). This, as some noted, made it difficult for staff to embed knowledge and skills due to the low volume of T1D pregnant women using HCL: “you have two or three months break [gap], and you go: ‘oh my God. What was it, that thing we said we must remember for this time?’” (HP-004). Some also suggested that this challenge would be compounded if several HCL systems “which are all slightly different” (HP-016) in terms of the training and support required, were available (Table 2).

### Healthcare professionals' training, resource, and support needs to rollout HCL

#### Training resources

To address the above challenges, interviewees proposed several solutions. To help ensure staff trained to support HCL use retained key skills and competencies, interviewees suggested making training resources, videos, and webinars available online, which healthcare professionals could (re)visit at a time of their choosing. They also recommended providing succinct checklists to limit “having to read a hundred and fifty-page user guide” (HP-003) (Table 2).

In keeping with the approach some described having adopted during the initial rollout of insulin pumps, some interviewees also discussed whether centers might begin by supporting pregnant women using one HCL system only (Table 2).

#### Infrastructure to support clinical teams to develop and consolidate local HCL expertise

When describing their trial experiences, interviewees praised the involvement of a research educator with CamAPS FX expertise who supported professionals at local sites when training pregnant women to use HCL (Table 2). Given their positive experiences, interviewees suggested that a similar approach be used to facilitate rollout, where individuals transitioning onto HCL received initial training from device manufacturers, replicating existing models to initiate insulin pump therapy (Table 2).

As an alternative, a minority proposed a hub-and-spoke model, “led from a central point” (HP-006), where women would receive training and glycemic support outsourced to “centers that are a bit more experienced supporting local teams … at least until [local] centers are more familiar with using the technologies” (HP-014).

#### Technical support for healthcare professionals

As with pump initiation in early pregnancy, healthcare professionals emphasized the need to invest time and resources during the first 2–4 weeks of HCL use. This included supporting women unfamiliar with component devices (e.g., assisting with set and sensor changes) or who “felt a bit nervous because they're having to trust the machine” (HP-009). All interviewees described how the ability to access technical, telephone support, which was “available on hand, 24/7” (HP-001) from the trial's research educator had been invaluable, especially when supporting women during this initial transition period (Table 2).

Hence, they suggested that if HCL technology was rolled out to pregnant women, staff would need access to a helpline, ideally on a 24 h basis, which offered similar technical support to that provided by the research educator, and which mirrored provisioning by pump and CGM manufacturers (Table 2).

#### Glycemic management support for healthcare professionals

Interviewees with limited or no prior experience of HCL use expressed a need, initially at least, for support to review and interpret women's HCL data, especially during the initial period after a woman had transitioned onto the system “to help people get the best out of it [HCL]” (HP-008). Specifically, interviewees noted that:

“there was a requirement to have … support around trying to interpret patterns, thinking about carb counting, thinking about where hypos come in… as the system begins to change its algorithm and zero in on particular insulin doses. And that, probably for all of these women, was a bit more intensive for the first two or three weeks” (HP-012).

As some interviewees observed, it took time and practice to develop skills to interpret data and determine which system settings to adjust: “you really only get the sense of the data … once you do it lots” (HP-013). Hence, some interviewees described benefiting from consulting and learning from experienced colleagues during the trial (Table 2):

Interviewees suggested potential ways to provide these kinds of support in routine clinical care. Reflecting on their use of the HCL's data sharing/remote access capabilities to obtain support from central trial staff, several, for instance, suggested that this functionality could enable inexperienced colleagues to seek mentorship/peer support from experienced staff at their own site or a regional expert:

“I think just having a mentor until you've got going… someone to ring up… who you share downloads with and say: this is what I did. What do you think? You know, I think that would be valuable.” (HP-015)

Some also suggested that this kind of support could be provided using an online portal where “you could drop the image of what you're unsure about, and then somebody who has expertise could perhaps either call you, if that's necessary, or could give feedback based on what they can see.” (HP-006). Several also described benefiting from intersite case-based meetings arranged to share learning and discuss challenging cases during the trial: “to hear about other people's experiences of what had gone wrong and what had gone well” (HP-002). To facilitate wider rollout, they suggested establishing closed-loop pregnancy “masterclass” (HP-013) meetings or workshops to help develop competence and confidence reviewing and interpreting data (Table 2).

### Short-term investment for long-term gains

While interviewees emphasized that wider rollout of HCL would require an initial investment of time and effort to train up staff to support technology use, most expressed optimism that, in the long-term, their workloads could lessen. As several explained, after optimizing settings, improvements in glycemic management when using the HCL could result in women requiring less support in the later stages of pregnancy: “if women are getting [more] time in range… through the technology doing it, we're probably going to have less input into micromanaging them” (HP-007). Others observed that women using HCL experienced fewer episodes of hypoglycemia, hence, “later support is hopefully less burdensome, if indeed there are fewer hypos and fewer overnight hypos” (HP-012).

Furthermore, healthcare professionals described how it was “a lot less onerous” (HP-004) to support women using HCL, because the automated regulation of basal insulin rates meant their focus was “almost all-around mealtime boluses and I don't have to think about the basal” (HP-006):

“if we could put the type 1's on the automatic pump, that would do at least some of the work for us… It's the CGM data that's the issue, looking at the CGM data. And that's why closed-loop is good because you're only looking at … the post meal bit. Whereas when you're looking at the CGM data for open loop and MDI you have to look at everything.” (HP-009)

Indeed, some speculated that, after making initial adjustments to settings/insulin-to-carbohydrate ratios, women using HCL might need less frequent or intensive glycemic reviews, although there would still be a need for someone to “eyeball the data” (HP-010). To this end, interviewees highlighted the potential value of using a system (similar to the CamAPS FX reports), which provided summary glycemic metrics for HCL users at each site/clinic. As interviewees explained, color-coded data enabled them to “interpret… a mountain of data within like five minutes” (HP-008) and quickly establish:

“what I need to know, across a clinic or a population base level. So it'll instantly highlight women who are above or below target, anyone who's having a ton of alarms, anything that's kind of out of the ordinary.” (HP-014)

As several further noted, using these kinds of reports had enabled them to “triage” individuals and identify who required support (Table 2).

## Discussion

This study reports healthcare professionals' views about the challenges and opportunities that could arise from rolling-out HCL technology to pregnant women with T1D. Our findings, alongside recommendations developed in our online workshop, offer practical ways to upskill professionals and potential ways to promote widespread and equitable access to this technology (see Box 4 for a summary of these recommendations).

**Box 4.** Summary of Recommendations

The recommendations presented below are ordered according to the priority attached to them by workshop participants.
• Ensure that healthcare professionals providing training and support to pregnant HCL users are proficient in the use of insulin pumps, CGM and HCL technology.
• Provide inexperienced centers/staff with support from device manufacturers to learn about insulin pump and CGM (if required) and train women transitioning onto a HCL.
• Provide healthcare professionals seeking to develop and/or refresh their skills with access to online webinars, videos, training resources, and succinct checklists, which are regularly updated and specific for using each closed-loop system.
• Support inexperienced sites/staff to review/interpret data by establishing regularly updated intra- and/or intersite meetings or ‘masterclasses' led by experienced healthcare professionals.
o Ensure that staff attending such meetings receive refresher training to take account of rapid developments in HCL technology.
• Provide 24 h technical support for healthcare professionals via a telephone helpline.
• Develop and implement a mentorship/peer support model to assist inexperienced centers/staff to review and interpret HCL users' glycemic and insulin data.
• Ensure that general hospital staff receive guidance to support pregnant HCL users who present acutely at emergency departments and maternity units. This could be provided by updating mandatory professional training modules (e.g., on Insulin Safety) to include:
o Information about HCL components (insulin pump, cannula, and CGM sensor) and where these might be located on a woman's body.
o Contact details and guidance on when to seek clinical input from local diabetes and obstetric specialists (e.g., during administration of corticosteroids, labor, and birth).
o Advice to follow local management protocols with expedited diabetes and obstetric review if a pregnant woman presents with ketones, with explicit guidance that HCL should not be used for management of DKA during pregnancy.

Interviewees emphasized that healthcare professionals providing training and support to pregnant HCL users would need to be proficient in the use of its constituent (CGM and insulin pump) technologies. They also raised concerns that existing disparities in insulin pump and CGM expertise^24–27^ might generate inequities in access to HCL technology. This is a valid concern given that others have identified professionals' lack of skills and competencies as being barriers to diabetes technology use more generally.^10,15,16,28^

Moreover, interviewees noted how infrequent exposure to small numbers of pregnant users might limit opportunities for staff to apply their skills and retain expertise, meaning that this issue could be more pronounced when supporting HCL use in T1D pregnancy. Interviewees, however, proposed a range of “low cost” solutions, such as making webinars, videos, training resources, and succinct checklists available online to help staff refresh their skills. These recommendations were endorsed by workshop participants who noted that several of these resources are already available for healthcare professionals supporting CamAPS FX users. Such training resources would need to be updated at regular intervals to keep abreast of rapidly changing HCL technology.

In keeping with interviewees' accounts, workshop participants also recommended that general hospital staff be given guidance to support HCL users who present acutely at emergency departments and maternity units. They suggested that existing mandatory training modules be updated to raise staffs' basic awareness and understanding of HCL technology and alert them to situations when they should contact diabetes specialists to seek clinical input (for further recommendations, see Box 4). In response to some interviewees' suggestion that only one HCL system initially be supported, workshop participants noted that this is not a feasible option because women are increasingly using different HCL systems before pregnancy. Participants noted that it would be unreasonable to ask existing users to switch systems and, in keeping with Sherr et al.'s recommendations,^29^ emphasized the need to respect users' choices and preferences.

To ensure equitable access via a national rollout, our findings suggest that it will be important to provide intensive, targeted initial support to inexperienced sites and staff. There was broad consensus among workshop participants that training to use the HCL's constituent components should be provided by device manufacturers; a similar recommendation has also recently been made by Sherr et al.^29^ Workshop participants also suggested that a central hub-and-spoke model (as used in the trial) would be unsuitable for real-world implementation. They expressed a strong preference for initial glycemic management after HCL initiation to be provided by local teams, with whom women had existing, trusting therapeutic relationships. With regard to interpreting HCL users' data and providing women with glycemic advice/support, interviewees valued opportunities to consult experienced colleagues, in particular (but not exclusively) during the initial weeks of HCL use.

To this end, workshop participants reinforced interviewees' suggestions by recommending local teams or individuals who lack experience reviewing and interpreting HCL data be offered mentorship/peer support from more experienced colleagues. They also recommended implementing interviewees' suggestions to develop collaborative models of support by establishing intra- and/or intersite case-based learning sessions or HCL pregnancy “masterclasses” led by experienced colleagues. However, workshop participants suggested that further consideration should be given to whether such support is provided locally, regionally, or nationally and by whom.

Moreover, they emphasized that funding may be needed to ensure that experienced staff have capacity to support less experienced colleagues/centers. Interviewees noted that such support could be provided virtually (by sharing HCL users' data with experienced colleagues); a model made logistically feasible by healthcare professionals' ability to access women's data and support glycemic management remotely.^30,31^ If a “masterclass” model is implemented, healthcare professionals would need regular refresher sessions to take account of rapid developments in HCL technology.

In line with interviewees' suggestions, workshop participants recommended that provision of HCL technology in routine care should include technical support for healthcare professionals, available via a 24 h telephone helpline, and similar to the support provided by insulin pump and CGM companies. Telephone support is already available for healthcare professionals supporting CamAPS FX users and as other HCL systems become available,^8^ similar technical support helplines will be needed. As workshop participants noted, these helplines would need to be able to offer bespoke recommendations for HCL use in T1D pregnancy.

While there may be some initial costs associated with developing the above training and support strategies, interviewees described how, in the long-term, workloads (and, hence, potentially costs) might be reduced because women using HCL might require less glycemic management input, especially during later pregnancy, than those using insulin pump or multiple daily injections regimens. This perspective mirrors that of healthcare professionals supporting adolescent HCL users^16^ and parents of young HCL users,^32,33^ who both described less need for healthcare professional input once initial adjustments to the system had been made.

Moreover, healthcare professionals in our study observed how email reports containing summary glycemic metrics had helped them to identify and triage individuals requiring additional support. Indeed, our findings suggest that using such metrics to triage caseloads could help address concerns raised in earlier studies that more time would be required during consultations to process and interpret large volumes of HCL data.^17^

### Strengths and limitations

This is the first study to consult healthcare professionals about the training and support needed to support a national rollout of HCL to pregnant women with T1D. The topicality and relevance of our findings have been enhanced by using a workshop at the end of the study to generate contemporary practical, implementable, and clinically relevant recommendations. A potential limitation is that our study took place within the context of a UK-based trial. Many participating centers had experience supporting insulin pump and CGM users, and some had HCL experience as a result of being involved in previous trials.

Hence, some interviewees may have been technology enthusiasts, with accordingly distinctive views about HCL rollout in T1D pregnancy, specific to a UK healthcare context, where there is universal CGM access. To offer a wider perspective on how a national rollout of HCL in T1D pregnancy could be achieved, future research could explore the perspectives of healthcare professionals working in less experienced centers or who have limited or no experience of supporting HCL users. Future research could also explore the experiences of healthcare professionals who support HCL users in countries that have different healthcare structures to those in the UK.

## Conclusions

Our recommendations offer important solutions and suggestions to address the challenges involved in rolling out HCL systems to pregnant women and are likely to have relevance to supporting rollout to other patient groups. A key priority will be to determine how best to develop mentorship services to support inexperienced staff delivering closed-loop therapy.

## Supplementary Material

Supplemental data
